# Data demonstrating the role of peroxiredoxin 2 as important anti-oxidant system in lung homeostasis

**DOI:** 10.1016/j.dib.2017.09.062

**Published:** 2017-09-30

**Authors:** Enrica Federti, Alessandro Matte, Alessandra Ghigo, Immacolata Andolfo, Cimino James, Angela Siciliano, Christophe Leboeuf, Anne Janin, Francesco Manna, Soo Young Choi, Achille Iolascon, Elisabetta Beneduce, Davide Melisi, Dae Won Kim, Sonia Levi, Lucia De Franceschi

**Affiliations:** a Dept. of Medicine, University of Verona-AOUI Verona, Verona, Italy; b Molecular Biotechnology Center and Department of Molecular Biotechnology and Health Science, University of Torino, Torino, Italy; c CEINGE and Dept. of Biochemistry, University of Naples, Naples, Italy; d Inserm, U1165, Paris F-75010, France; e Université Paris 7- Denis Diderot, Paris, France; f AP-HP, Hôpital Saint-Louis, F-75010 Paris, France; g Institute of Bioscience and Biotechnology, Hallym University, Gangowo-do, Republic of Korea; h Division of Neuroscience, San Raffaele Scientific Institute, Milano, Italy; i Vita-Salute San Raffaele University, Milano, Italy

## Abstract

The data presented in this article are related to the research paper entitled “peroxiredoxin-2 plays a pivotal role as multimodal cytoprotector in the early phase of pulmonary hypertension” (Federti et al., 2017) [1]. Data show that the absence of peroxiredoxin-2 (Prx2) is associated with increased lung oxidation and pulmonary vascular endothelial dysfunction. Prx2^−/^^−^ mice displayed activation of the redox-sensitive transcriptional factors, NF-kB and Nrf2, and increased expression of cytoprotective system such as heme-oxygenase-1 (HO-1). We also noted increased expression of both markers of vascular activation and extracellular matrix remodeling. The administration of the recombinant fusion protein PEP Prx2 reduced the activation of NF-kB and Nrf2 and was paralleled by a decrease in HO-1 and in vascular endothelial abnormal activation. Prolonged hypoxia was used to trigger pulmonary artery hypertension (PAH). Prx2^−/^^−^ precociously developed PAH compared to wildtype animals.

**Specifications Table**

**Table:** 

Subject area	*Health Sciences*
More specific subject area	*Oxidation, peroxiredoxin-2 and pulmonary artery hypertension*
Type of data	*Text file, Figures*
How data was acquired	Image Quant Las Mini 4000 Digital Imaging System (GE Healthcare Life Sciences). Densitometric analyses were performed using the ImageQuant TL software (GE Healthcare Life Sciences).
Data format	*Raw analyzed*
Experimental factors	*C57B6/2J as wildtype mice and Prx2^−/^^−^ mice*
Experimental features	*Protein expression was analyzed by Western-blotting.*
	Oxidized proteins were revealed by the Oxyblot Protein Oxidation Detection Kit (EMD Millipore); MDA pulmonary levels were evaluated by Oxiselect MDA Immunoblot kit (GE Healthcare).
Data source location	*Dept. of Medicine, LURM, Policlinico GB Rossi, University of Verona and AOUI Verona; Verona; Italy*
Data accessibility	*Data are available with this article*

**Value of the data**

• Our data show that the absence of Prx2 is associated with increased lung oxidation and abnormal pulmonary vascular leakage.
• Treatment with fusion protein PEP Prx2 prevents the activation of redox related transcriptional factors and modulates anti-oxidant systems in both wildtype and Prx2^−/−^ mice.
• PEP Prx2 significantly reduces protein oxidation in lung from exposed to prolonged hypoxia used to trigger pulmonary artery hypertension.

## 1 Data

**Fig. 1:**
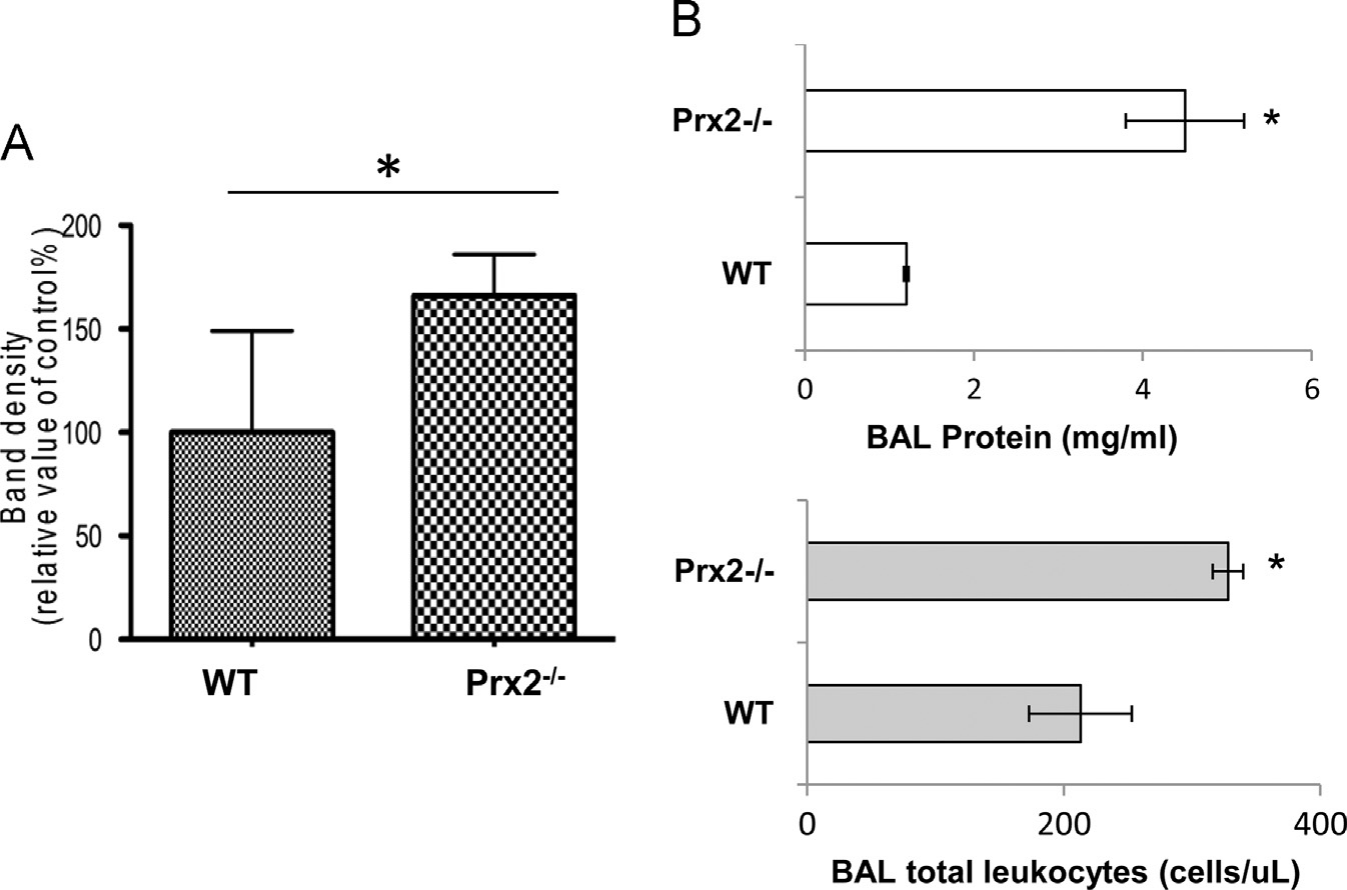
**A.** 10 μg of soluble proteins of lung homogenate were tested for MDA-protein adducts. Quantification of band area was performed by densitometry and expressed as % of WT. The data are presented as means ± SD of at least three independent experiments; statistically significant differences were determined by Student's t-test. *p < 0.05. **B. Upper panel.** BAL protein content from wildtype (WT) and Prx2^−^^/^^−^ mice under normoxic condition. Data are presented as means± SD (*n* = 6; *p < 0.05 compared to WT mice). **Lower panel.** BAL leukocyte content from wildtype (WT) and Prx2^−^^/^^−^ mice under normoxic condition. Data are presented as means ± SD (*n* = 6; *p < 0.05 compared to WT mice).

**Fig. 2:**
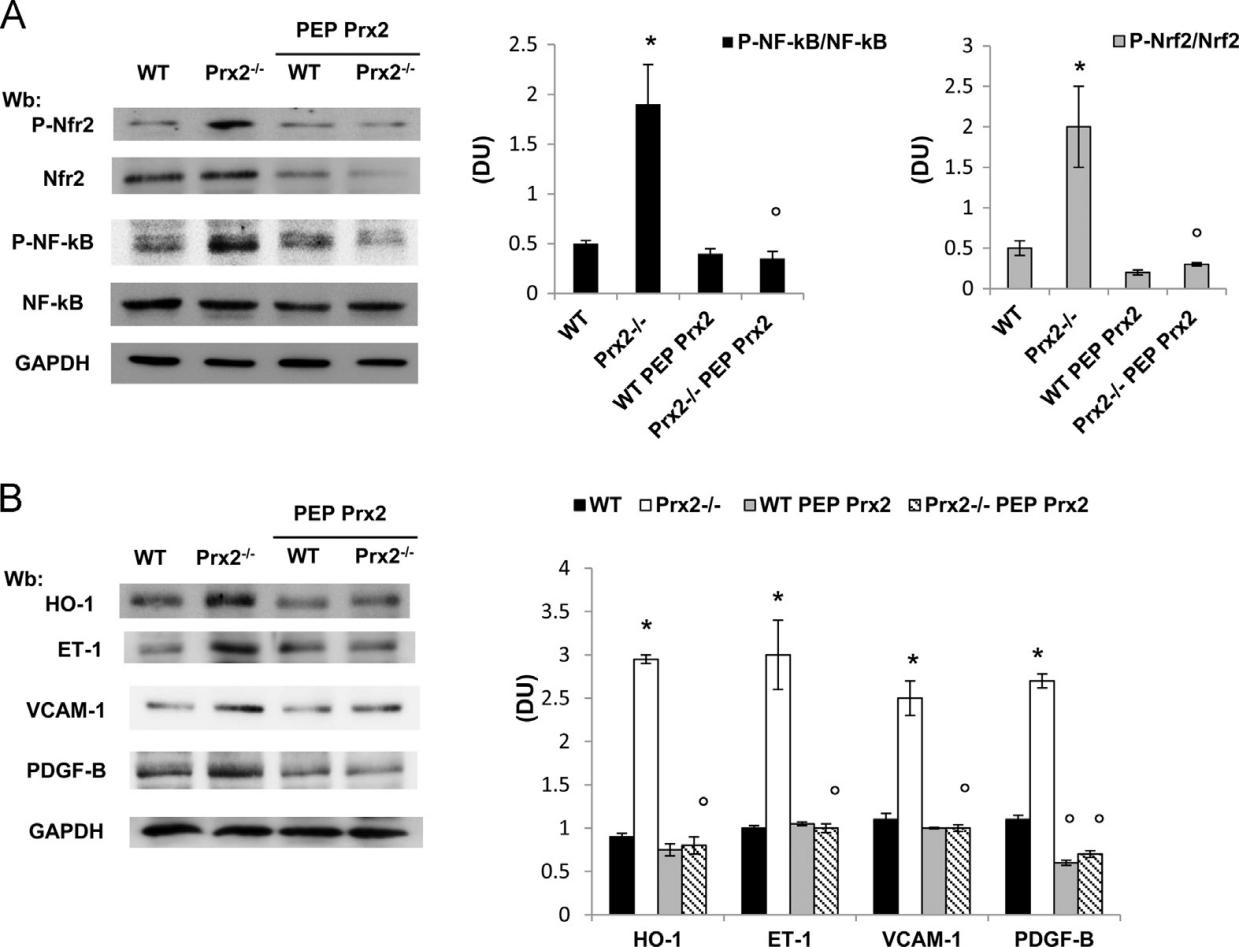
**A.** Immunoblot analysis with specific antibodies against phospho-Nrf2 (P-Nrf2), Nrf2 phospho-NF-kB (P-NF-kB) and NF-kB of lung from wildtype (WT) and Prx2^−^^/^^−^ mice under normoxic condition. One representative gel from six with similar results is presented. **Right panel.** Relative quantification of immunoreactivity (DU: Density Units) of phospho-NF-kB (P-NF-kB), NF-k, phospho-Nrf2 (P-Nrf2) and Nrf2 of lung from wildtype (WT) and Prx2^−^^/^^−^ mice under normoxic condition treated with either vehicle or penetrating peptide fusion protein peroxiredoxin-2 (PEP Prx2). Data are shown as means ± SD (*n* = 6). *p < 0.05 compared to wildtype; °*p* < 005 compared to vehicle treated mice. **B.** Immunoblot analysis with specific antibodies against heme-oxygenase 1 (HO-1), endothelin-1 (ET-1), vascular cell adhesion molecule-1 (VCAM-1), under normoxic condition treated with either vehicle or penetrating peptide fusion protein peroxiredoxin-2 (PEP Prx2). One representative gel from six with similar results is presented. **Right panel.** Relative quantification of immunoreactivity (DU: Density Units) of heme-oxygenase 1 (HO-1), endothelin-1 (ET-1), vascular cell adhesion molecule-1 (VCAM-1), platelet derived growth factor-B (PDGF-B) of lung from wildtype (WT) and Prx2^−^^/^^−^ mice under normoxic condition treated with either vehicle or penetrating peptide fusion protein peroxiredoxin-2 (PEP Prx2). Data are shown as means ± SD (*n* = 6). *p < 0.05 compared to wildtype; °*p* < 0.05 compared to vehicle treated mice.

Data show increased lung oxidation (Fig. 1A) and abnormal pulmonary vascular leakage in the absence of Prx2 (Fig. 1B). This was paralleled by the activation of redox-sensitive transcriptional factors NF-kB and Nrf2 in lung from Prx2^−/−^ compared to wildtype animals (Fig. 2A). Indeed, in Prx2^−/−^ we observed (i) increased expression of heme-oxygenase 1 (HO-1), a Nrf2 related cytoprotective system; (ii) markers of vascular endothelial activation such as endothelin-1 (ET-1) and vascular cell adhesion molecule -1 (VCAM-1) and (iii) marker of extracellular matrix remodeling as the platelet growth factor- B (PDGF-B) that has been recently function linked to the development of pulmonary artery hypertension (Fig. 2B). To verify the role of Prx2 as important anti-oxidant system in pulmonary homeostasis, we administrated the recombinant fusion protein PEP Prx2 at the dosage of 3 mg/Kg/d ip or vehicle for 4 weeks [1], [2], [3]. As shown in Fig. 2, PEP Prx2 significantly reduced both NF-kB and Nrf2 activation in lung from Prx2^−/−^ and decreased the expression of both HO-1 and markers of vascular endothelial activation or extracellular matrix remodeling.

Using prolonged hypoxia to trigger pulmonary artery hypertension, we observed severe lung pathologic damage and the precocious development of pulmonary artery hypertension in Prx2^−^^/^^−^ mice compared to wildtype animals [4], [5], [6]. This was associated with (i) marked activation of redox- related transcriptional factors; (ii) severe endoplasmic-reticulum stress with activation of the unfolded protein response (UPR) system; and (iii) activation of autophagy [1].

**Fig. 3:**
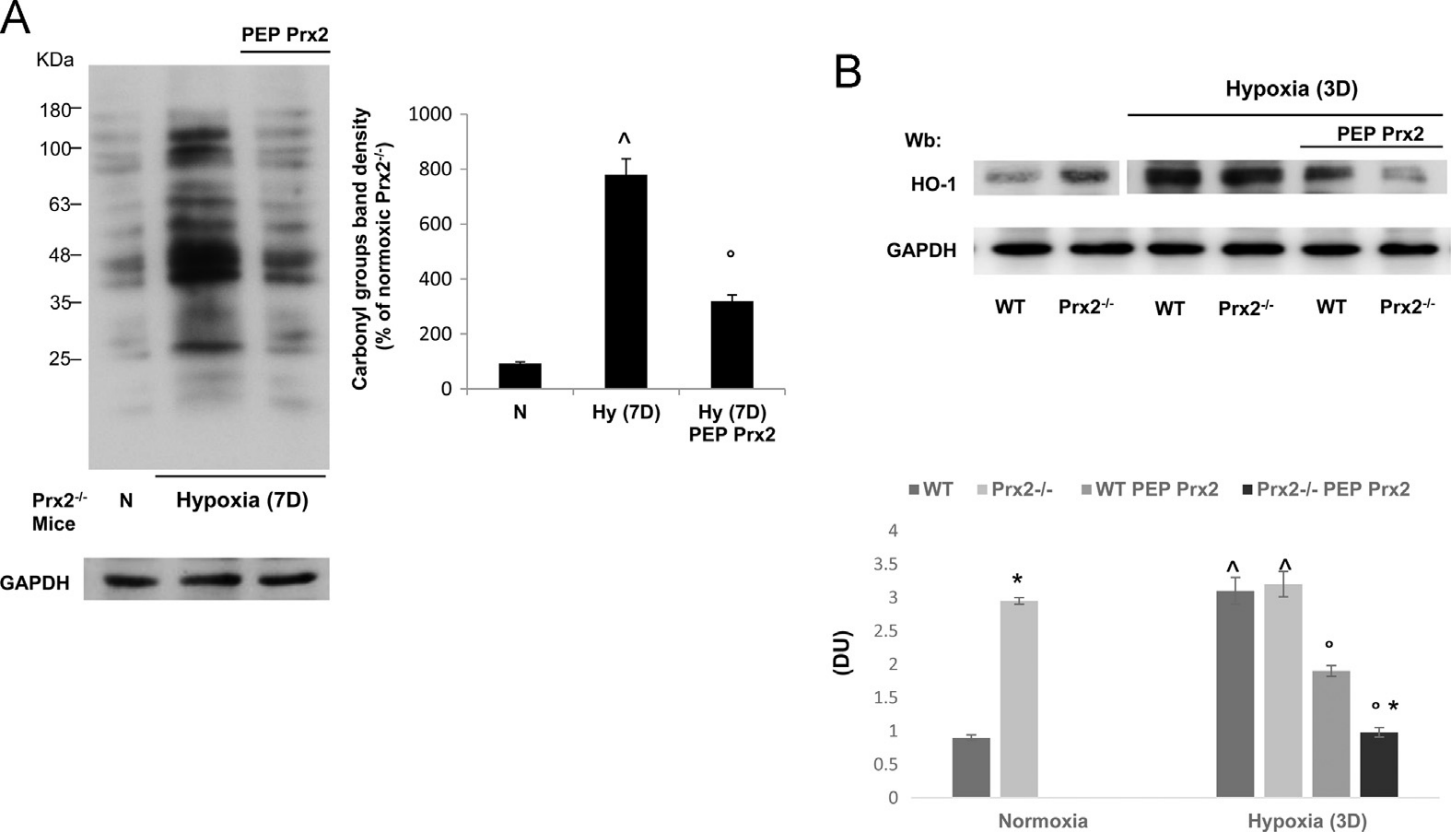
**A.** The carbonylated proteins (1 ug) from lung of Prx2^−^^/^^−^ mice under normoxic condition or exposed to 7 days (7D) hypoxia/reoxygenation stress (H/R) treated with either vehicle or penetrating peptide fusion protein peroxiredoxin-2 (PEP Prx2) at the dosage of 3 mg/Kg/d ip or vehicle for 4 weeks before and during hypoxia (8% oxygen for 7 days). **Right panel.** Quantification of band area was performed by densitometry and expressed as % of Prx2 under normoxia. The data are presented as means ± SD of at least three independent experiments; ^*p* < 0.05 compared to Prx2^−^^/^^−^ normoxic mice; °*p* < 005 compared to vehicle treated mice. (*n* = 3). **B.** Immunoblot analysis with specific antibodies against heme-oxygenase-1 (HO-1) of lung from wildtype (WT) and Prx2^−^^/^^−^ mice under normoxic condition or exposed to 3 days (3D) hypoxia/reoxygenation stress (H/R) treated with either vehicle or penetrating peptide fusion protein peroxiredoxin-2 (PEP Prx2) at the dosage of 3 mg/Kg/d ip or vehicle for 4 weeks before and during hypoxia (8% oxygen for 3 days). One representative gel from five with similar results is presented. **Lower panel.** Relative quantification of immunoreactivity (DU: Density Units) of heme-oxygenase 1 of lung from wildtype (WT) and Prx2^−^^/^^−^ mice under normoxic condition or exposed to 3 days (3D) hypoxia/reoxygenation stress (H/R) treated with either vehicle or PEP Prx2. Data are presented as means ± SD of at least five independent experiments; *p < 0.05 compared to wildtype; ^*p* < 0.05 compared to Prx2^−^^/^^−^ normoxic mice; °*p* < 005 compared to vehicle treated mice. (*n* = 5).

PEP Prx2 treatment prevented the hypoxia induced protein oxidation in mice exposed to prolonged hypoxia (7 days; Fig. 3A) and reduced the hypoxia induced increased expression of HO-1 in both mouse strains exposed to 3 days hypoxia (Fig. 3B). Collectively, these data indicate the important role of Prx2 in lung homeostasis against hypoxia, a known trigger of lung injury.

## 2 Experimental design, materials and methods

### 2.1 MDA assay

MDA was determined as previously reported [7], [8].

### 2.2 Measurement of BAL protein content

#### 2.2.1 Bronchoalveolar lavage assay

Bronchoalveolar lavage (BAL) fluids were collected and cellular contents were recovered by centrifugation and counted by microcytometry as previously reported [6], [9].

#### 2.2.2 Immunoblot analysis

Frozen lung from each studied group were homogenized and lysed with iced lyses buffer as previously described [3], [10], [11]. Gels were transferred to nitrocellulose membranes for immuno-blot analysis with specific antibody: anti-NFkB-phospho-S536 (93H1) (Cell Signaling Technology, Leiden, NL); anti-NFkB p65 (C22B4) (Cell Signaling Technology, Leiden, NL); anti-Nrf2-phospho-S40 (Clone EP1809Y, AbCam, Cambridge, UK); anti-Nrf2 (AbCam, Cambridge, UK); anti-Heme Oxygenase-1 (HO-1) (Santa Cruz Biotechnology, Heidelberg, Germany), anti-Endothelin-1 (ET-1) (Santa Cruz Biotechnology, Heidelberg, Germany); anti-VCAM-1 (R and D Systems, Minneapolis, MN, USA); anti-PDGF-B (AbCam, Cambridge, UK); anti-GAPDH (Sigma Aldrich, Saint Louis, MO, USA) was used as loading control. Images were acquired using Image Quant Las Mini 4000 Digital Imaging System (GE Healthcare Life Sciences). Densitometric analyses were performed using the ImageQuant TL software (GE Healthcare Life Sciences) [11].

#### 2.2.3 Measurement of lung protein oxidation

Oxidized proteins were revealed by the Oxyblot Protein Oxidation Detection Kit (EMD Millipore). In brief, the soluble protein extracts were derivatized to 2,4-dinitrophenylhydrazone (DNP) and 1 µg was loaded on 12% SDS-PAGE, blotted and incubated with an anti-DNP antibody, followed by an HRP conjugated secondary antibody. The bound activity was revealed by ECL (GE Healthcare). Oxidized proteins were revealed by the Oxyblot Protein Oxidation Detection Kit (EMD Millipore). In brief, the soluble protein extracts were derivatized to 2,4-dinitrophenylhydrazone (DNP) and 1 ug was loaded on 12% SDS-PAGE, blotted and incubated with an anti-DNP antibody, followed by an HRP conjugated secondary antibody. The bound activity was revealed by ECL (GE Healthcare) [12], [13], [14].

#### 2.2.4 Generation of recombinant-PEP Prx2 fusion protein (PEP Prx2)

The fusion protein PEP Prx2 was generated as previously reported [2], [3].
