# Supplementary material for: Data demonstrating the role of peroxiredoxin 2 as important anti-oxidant system in lung homeostasis
Source: Data Brief. 2017 Sep 30;15:376–81. doi: 10.1016/j.dib.2017.09.062 (PMC5636020; doi:10.1016/j.dib.2017.09.062)
Supplement: Supplementary file 1 — Supplementary material [file mmc1.docx]

The Authors have no conflict to declare.
